# Definition and reporting of pilot and feasibility studies

**DOI:** 10.1186/1745-6215-14-S1-O18

**Published:** 2013-11-29

**Authors:** Sandra Eldridge, Christine Bond, Mike Campbell, Gill Lancaster, Lehana Thabane, Sally Hopwell

**Affiliations:** 1 Queen Mary University of London, London, UK; 2 University of Aberdeen, Aberdeen, UK; 3 University of Sheffield, Sheffield, UK; 4 University of Lancaster, Lancaster, UK; 5 McMaster University, Hamilton, Canada; 6 University of Oxford, Oxford, UK

## 

Pilot/feasibility studies can be an essential part of trial preparation, particularly in planning complex interventions. However, recent research indicates that these studies suffer from publication bias and a lack of clarity in the objectives and methodological focus. Misunderstandings about the purpose of pilot/feasibility studies mean that opportunities to answer the important research questions at the piloting/feasibility stage may be lost. As a result full trials may be less efficient, interventions less effective, and trials may run into serious problems with conduct that could have been avoided with proper piloting. NIHR have produced definitions of feasibility and pilot studies to try and address some of these issues.

Nevertheless, there remains considerable interest and debate in this area and further guidelines are needed. We are currently producing CONSORT reporting guidelines for feasibility and pilot studies conducted in advance of a full trial. These guidelines include clarification of definitions. Between July and October 2013 we are conducting a Delphi consensus study on draft guidelines on 100 participants. We will present the background to this work, new ideas about the definition of feasibility and pilot studies and a summary of some of the issues that have arisen in trying to construct the guidelines, for example, what should be reported in relation to the main trial, how bias, blinding and multiple objectives should be handled, whether cost-effectiveness analyses are justified in a pilot study. In a later session after the end of the official conference we will present the guidelines in full for further discussion.

